# Supplementary material for: The Effects of Stress on Hippocampal Neurogenesis and Behavior in the Absence of Lipocalin-2
Source: Int J Mol Sci. 2023 Oct 24;24(21):15537. doi: 10.3390/ijms242115537 (PMC10649401; doi:10.3390/ijms242115537)
Supplement: Supplementary file 1 [file ijms-24-15537-s001.zip › ijms-2626532-supplementary.pdf]

Supplementary Figure S1

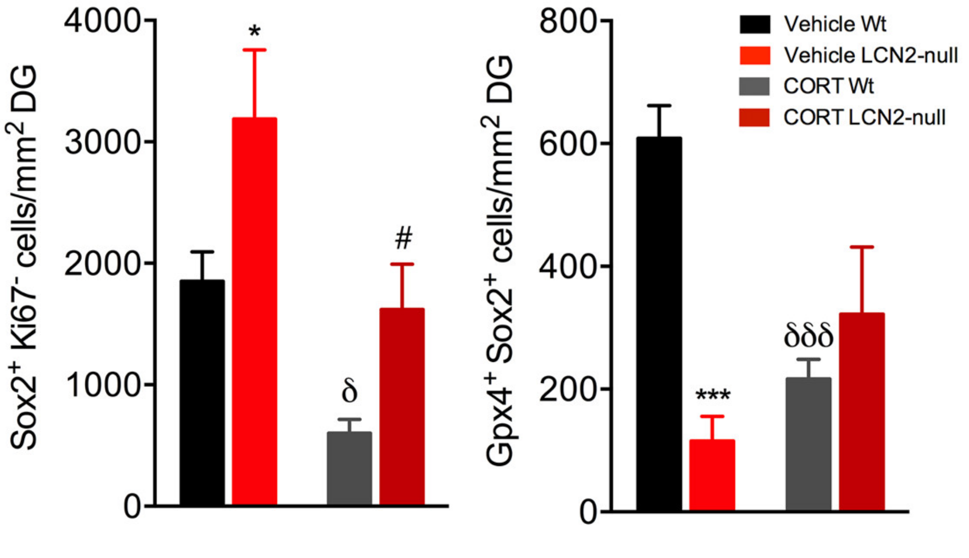

Figure S1: Chronic CORT administration reduces type-1 nonradial stem cells. Quantification of nonradial Ki67$^-$ Sox2$^+$ type-1 stem cells, after chronic CORT exposure, revealed a significant effect on this population, regardless of the animals' genotype. Data are presented as mean ± SEM and were analyzed by two-way ANOVA with Bonferroni's multiple comparison test. *Denotes differences between vehicle Wt and LCN2-null mice; $^{\delta}$between vehicle and CORT Wt; $^{\#}$between vehicle and CORT LCN2-null mice. $^{*,\#,\delta}p \leq 0.05$. ***, δδδ $p \leq 0.001$.
